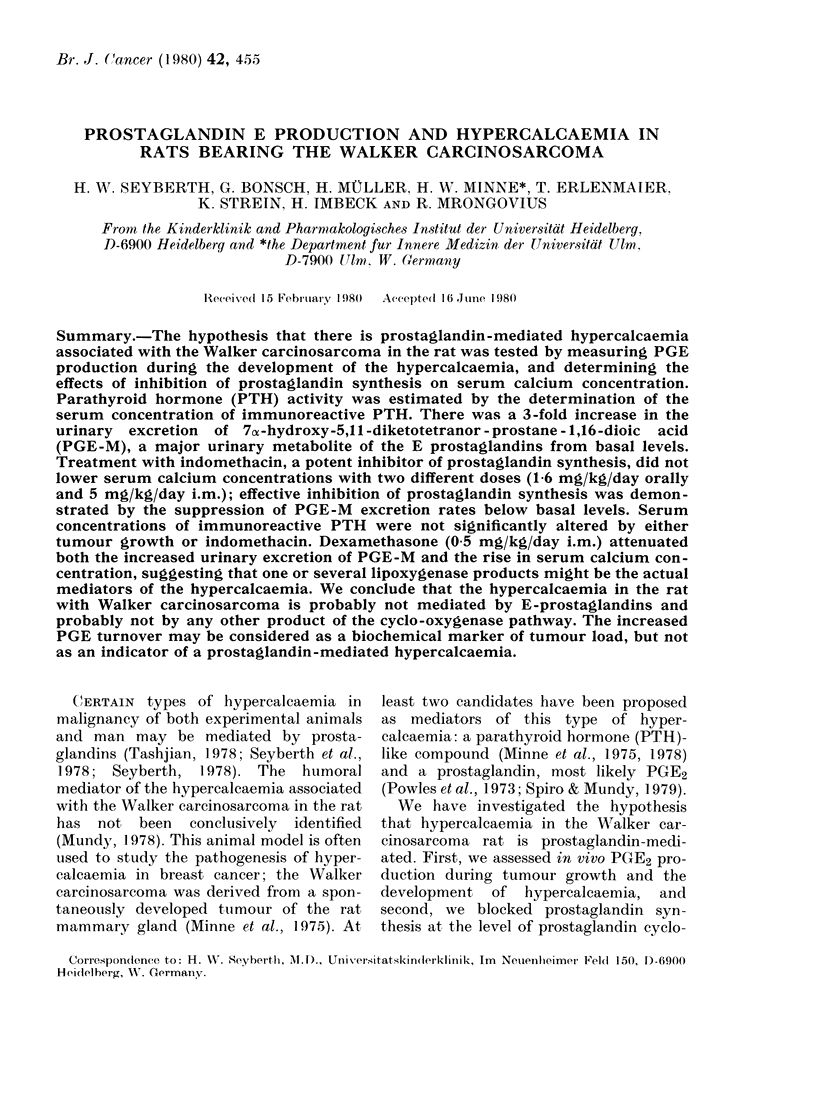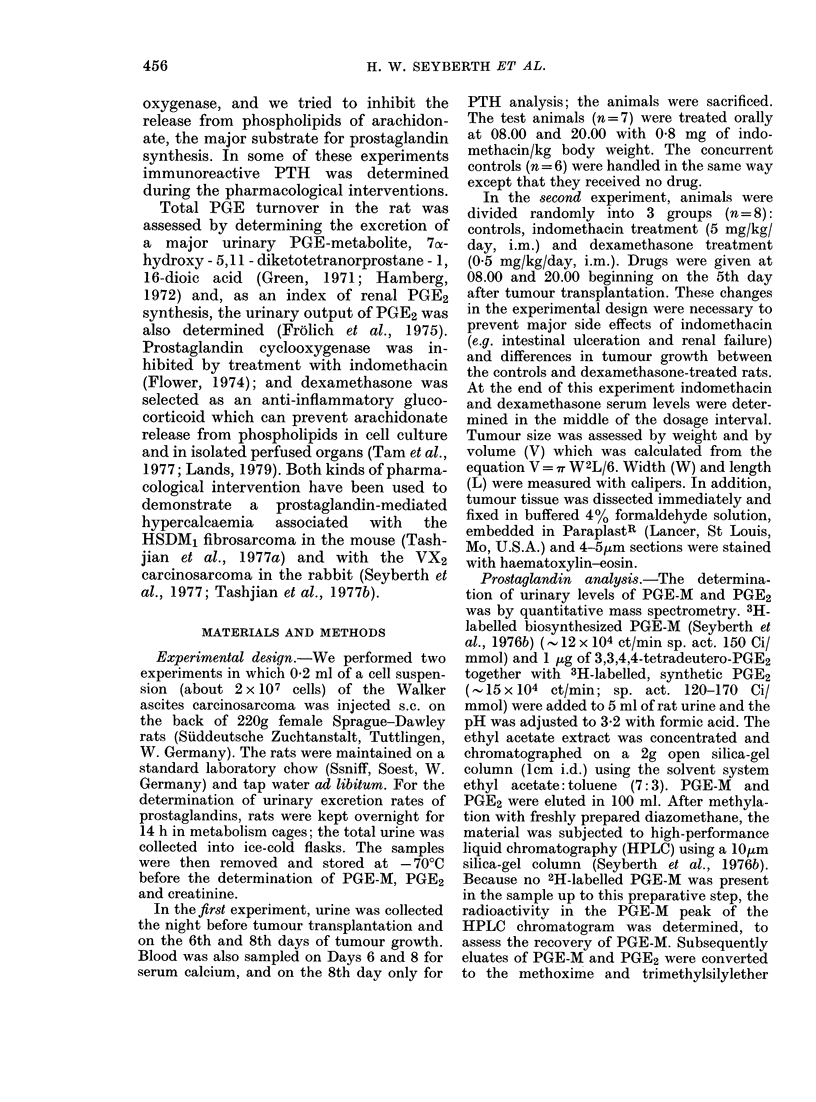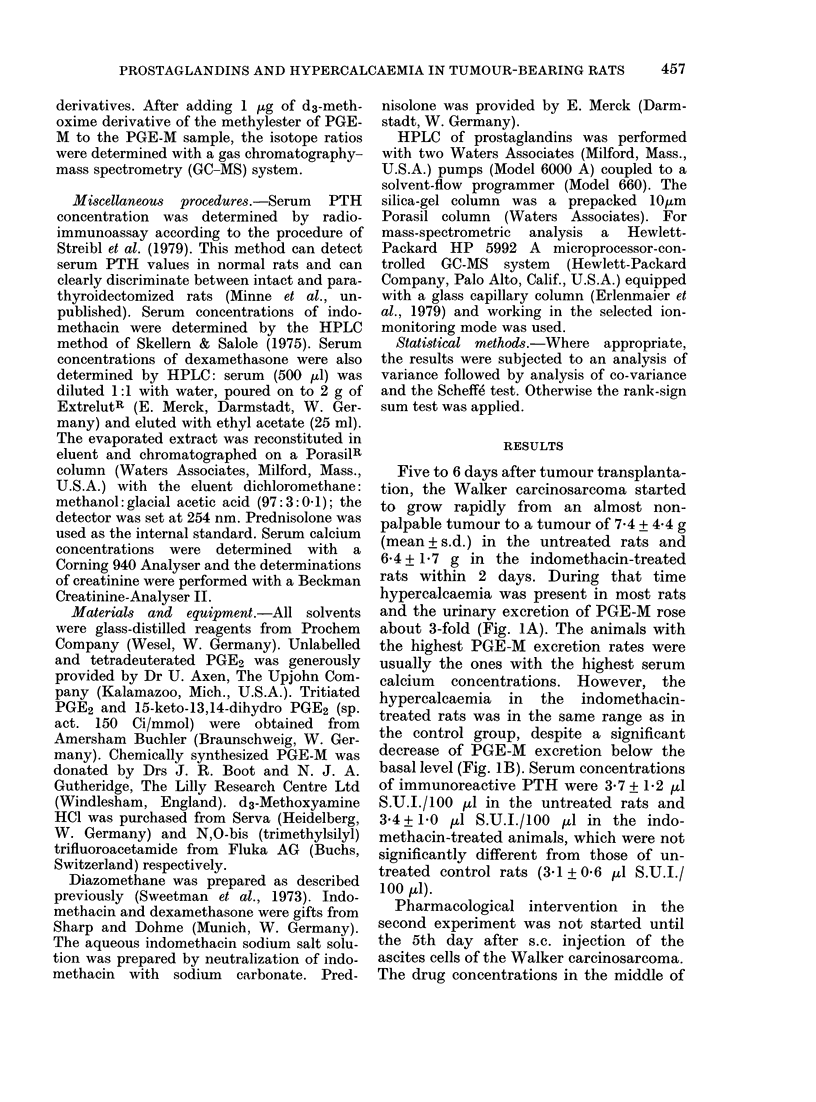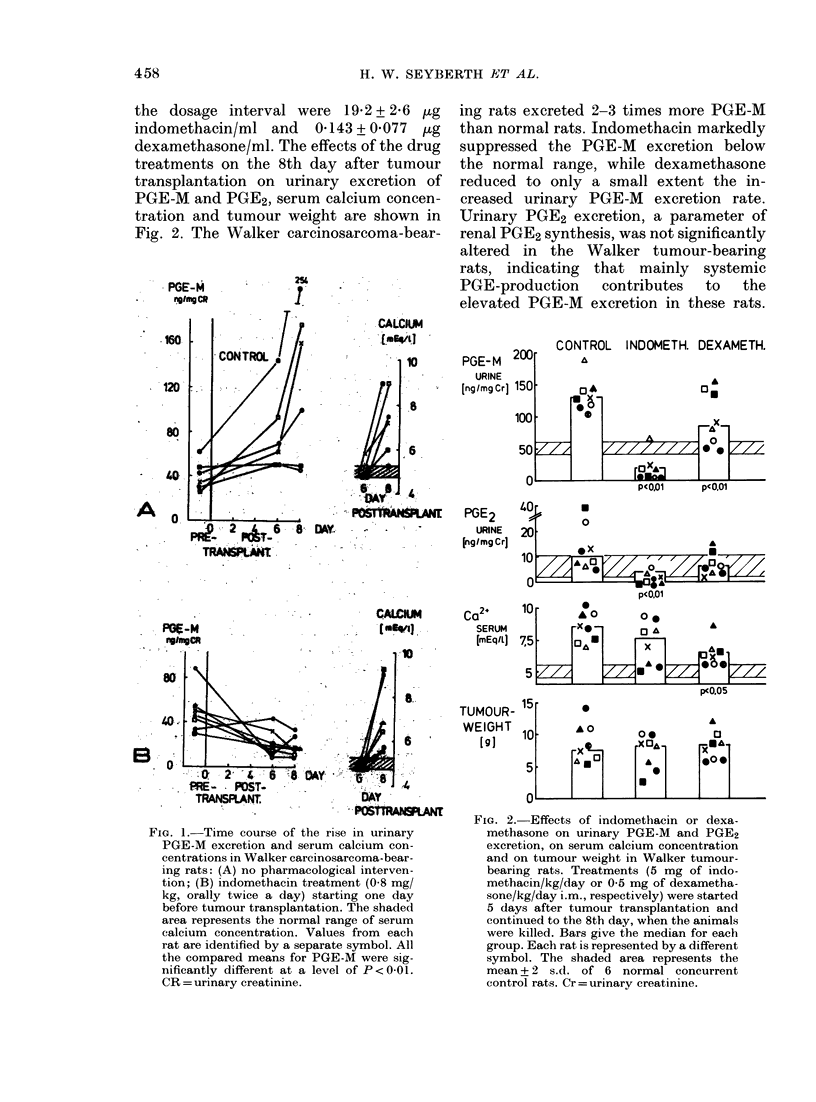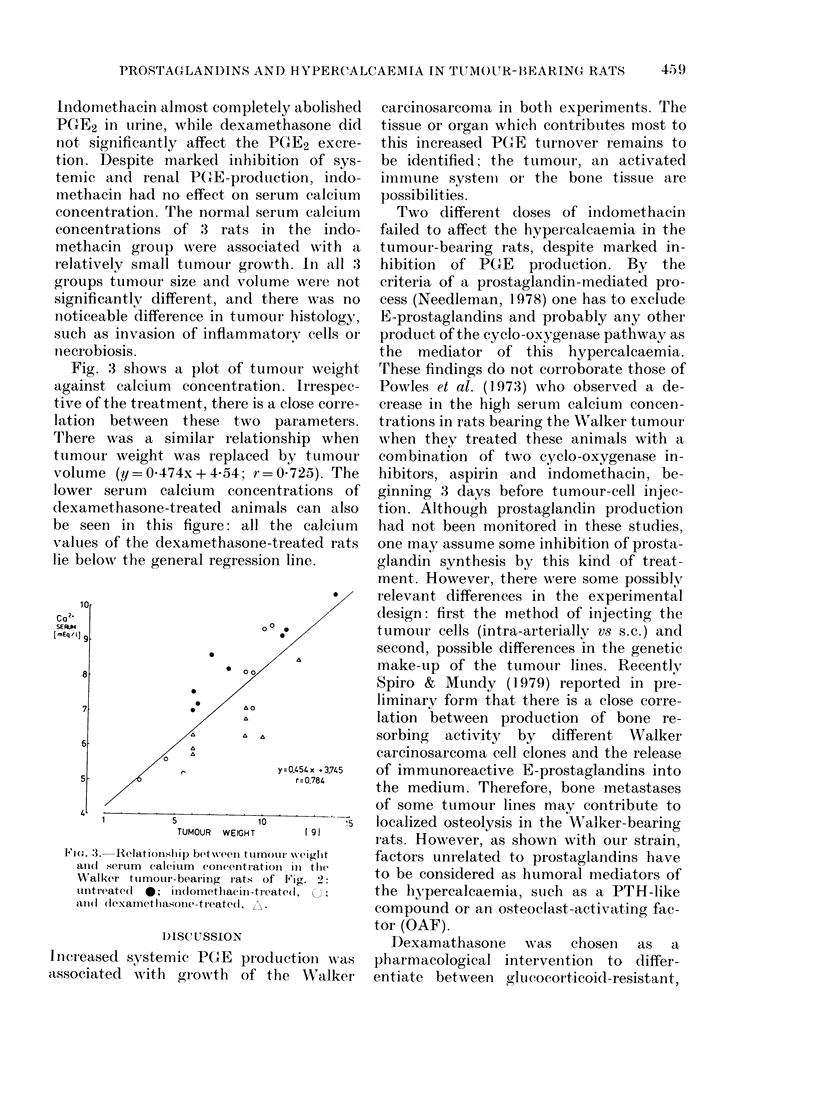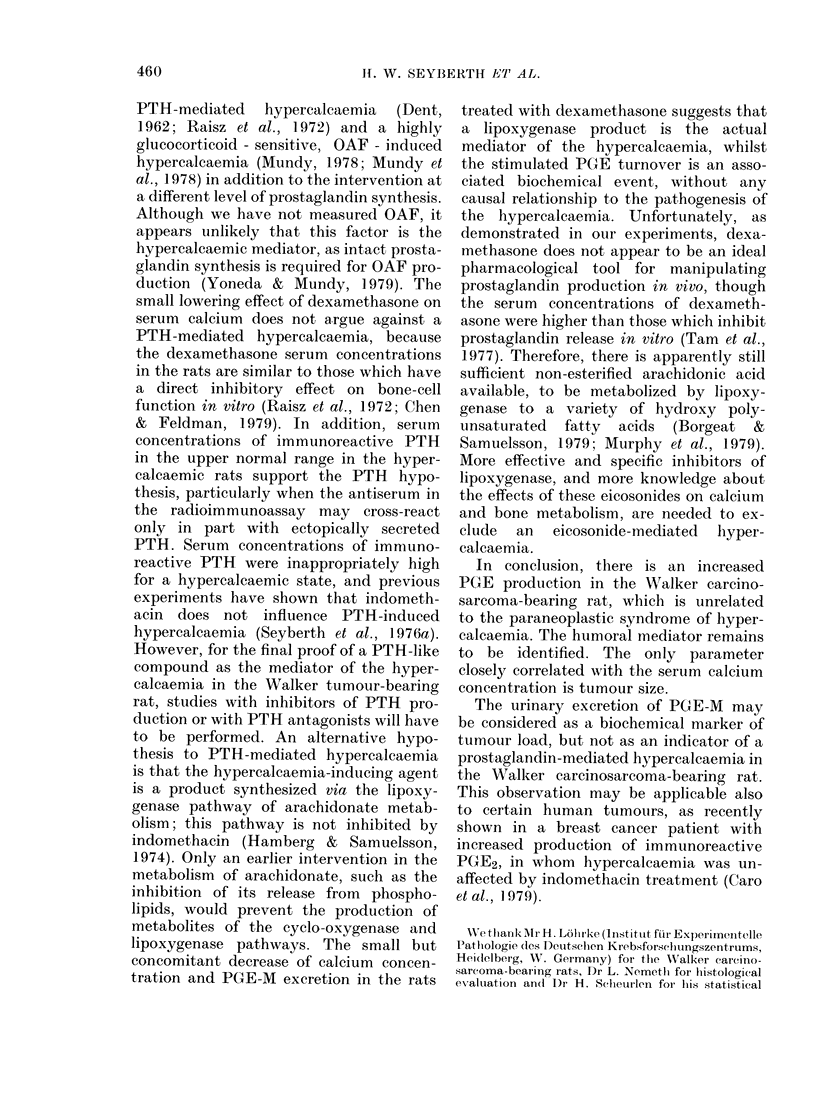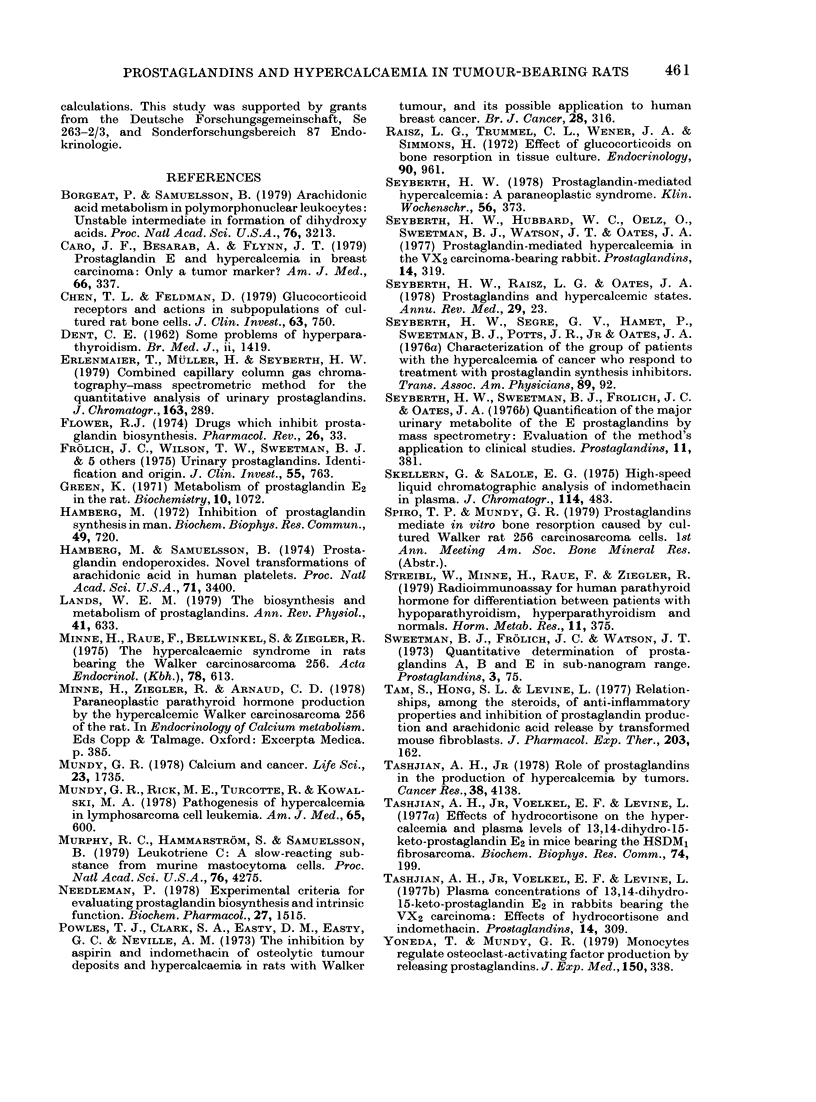# Prostaglandin E production and hypercalcaemia in rats bearing the Walker carcinosarcoma

**DOI:** 10.1038/bjc.1980.258

**Published:** 1980-09

**Authors:** H. W. Seyberth, G. Bonsch, H. Müller, H. W. Minne, T. Erlenmaier, K. Strein, H. Imbeck, R. Mrongovius

## Abstract

The hypothesis that there is prostaglandin-mediated hypercalcaemia associated with the Walker carcinosarcoma in the rat was tested by measuring PGE production during the development of the hypercalcaemia, and determining the effects of inhibition of prostaglandin synthesis on serum calcium concentration. Parathyroid hormone (PTH) activity was estimated by the determination of the serum concentration of immunoreactive PTH. There was a 3-fold increase in the urinary excretion of 7α-hydroxy-5,11-diketotetranor-prostane-1,16-dioic acid (PGE-M), a major urinary metabolite of the E prostaglandins from basal levels. Treatment with indomethacin, a potent inhibitor of prostaglandin synthesis, did not lower serum calcium concentrations with two different doses (1·6 mg/kg/day orally and 5 mg/kg/day i.m.); effective inhibition of prostaglandin synthesis was demonstrated by the suppression of PGE-M excretion rates below basal levels. Serum concentrations of immunoreactive PTH were not significantly altered by either tumour growth or indomethacin. Dexamethasone (0·5 mg/kg/day i.m.) attenuated both the increased urinary excretion of PGE-M and the rise in serum calcium concentration, suggesting that one or several lipoxygenase products might be the actual mediators of the hypercalcaemia. We conclude that the hypercalcaemia in the rat with Walker carcinosarcoma is probably not mediated by E-prostaglandins and probably not by any other product of the cyclo-oxygenase pathway. The increased PGE turnover may be considered as a biochemical marker of tumour load, but not as an indicator of a prostaglandin-mediated hypercalcaemia.


*Br. J. Cancer* (1980) **42**, 455

### PROSTAGLANDIN E PRODUCTION AND HYPERCALCAEMIA IN RATS BEARING THE WALKER CARCINOSARCOMA

H. W. SEYBERTH, G. BONSCH, H. MÜLLER, H. W. MINNE*, T. ERLENMAIER, K. STREIN, H. IMBECK AND R. MRONGOVIUS

*From the Kinderklinik and Pharmakologisches Institut der Universität Heidelberg, D-6900 Heidelberg and *the Department fur Innere Medizin der Universität Ulm, D-7900 Ulm, W. Germany*

Received 15 February 1980 Accepted 16 June 1980

**Summary.—The hypothesis that there is prostaglandin-mediated hypercalcaemia associated with the Walker carcinosarcoma in the rat was tested by measuring PGE production during the development of the hypercalcaemia, and determining the effects of inhibition of prostaglandin synthesis on serum calcium concentration. Parathyroid hormone (PTH) activity was estimated by the determination of the serum concentration of immunoreactive PTH. There was a 3-fold increase in the urinary excretion of 7α-hydroxy-5,11-diketotetranor-prostane-1,16-dioic acid (PGE-M), a major urinary metabolite of the E prostaglandins from basal levels. Treatment with indomethacin, a potent inhibitor of prostaglandin synthesis, did not lower serum calcium concentrations with two different doses (1·6 mg/kg/day orally and 5 mg/kg/day i.m.); effective inhibition of prostaglandin synthesis was demonstrated by the suppression of PGE-M excretion rates below basal levels. Serum concentrations of immunoreactive PTH were not significantly altered by either tumour growth or indomethacin. Dexamethasone (0·5 mg/kg/day i.m.) attenuated both the increased urinary excretion of PGE-M and the rise in serum calcium concentration, suggesting that one or several lipoxygenase products might be the actual mediators of the hypercalcaemia. We conclude that the hypercalcaemia in the rat with Walker carcinosarcoma is probably not mediated by E-prostaglandins and probably not by any other product of the cyclo-oxygenase pathway. The increased PGE turnover may be considered as a biochemical marker of tumour load, but not as an indicator of a prostaglandin-mediated hypercalcaemia.**

CERTAIN types of hypercalcaemia in malignancy of both experimental animals and man may be mediated by prostaglandins (Tashjian, 1978; Seyberth *et al.*, 1978; Seyberth, 1978). The humoral mediator of the hypercalcaemia associated with the Walker carcinosarcoma in the rat has not been conclusively identified (Mundy, 1978). This animal model is often used to study the pathogenesis of hypercalcaemia in breast cancer; the Walker carcinosarcoma was derived from a spontaneously developed tumour of the rat mammary gland (Minne *et al.*, 1975). At least two candidates have been proposed as mediators of this type of hypercalcaemia: a parathyroid hormone (PTH)-like compound (Minne *et al.*, 1975, 1978) and a prostaglandin, most likely $PGE_2$ (Powles *et al.*, 1973; Spiro & Mundy, 1979).

We have investigated the hypothesis that hypercalcaemia in the Walker carcinosarcoma rat is prostaglandin-mediated. First, we assessed *in vivo* $PGE_2$ production during tumour growth and the development of hypercalcaemia, and second, we blocked prostaglandin synthesis at the level of prostaglandin cyclo-

Correspondence to: H. W. Seyberth, M.D., Universitatskinderklinik, Im Neuenheimer Feld 150, D-6900 Heidelberg, W. Germany.

oxygenase, and we tried to inhibit the release from phospholipids of arachidonate, the major substrate for prostaglandin synthesis. In some of these experiments immunoreactive PTH was determined during the pharmacological interventions.

Total PGE turnover in the rat was assessed by determining the excretion of a major urinary PGE-metabolite, 7α-hydroxy-5,11-diketotetranorprostane-1, 16-dioic acid (Green, 1971; Hamberg, 1972) and, as an index of renal $PGE_2$ synthesis, the urinary output of $PGE_2$ was also determined (Frölich *et al.*, 1975). Prostaglandin cyclooxygenase was inhibited by treatment with indomethacin (Flower, 1974); and dexamethasone was selected as an anti-inflammatory glucocorticoid which can prevent arachidonate release from phospholipids in cell culture and in isolated perfused organs (Tam *et al.*, 1977; Lands, 1979). Both kinds of pharmacological intervention have been used to demonstrate a prostaglandin-mediated hypercalcaemia associated with the $HSDM_1$ fibrosarcoma in the mouse (Tashjian *et al.*, 1977*a*) and with the $VX_2$ carcinosarcoma in the rabbit (Seyberth *et al.*, 1977; Tashjian *et al.*, 1977*b*).

### MATERIALS AND METHODS

*Experimental design.*—We performed two experiments in which 0·2 ml of a cell suspension (about $2 \times 10^7$ cells) of the Walker ascites carcinosarcoma was injected s.c. on the back of 220g female Sprague–Dawley rats (Süddeutsche Zuchtanstalt, Tuttlingen, W. Germany). The rats were maintained on a standard laboratory chow (Ssniff, Soest, W. Germany) and tap water *ad libitum*. For the determination of urinary excretion rates of prostaglandins, rats were kept overnight for 14 h in metabolism cages; the total urine was collected into ice-cold flasks. The samples were then removed and stored at −70°C before the determination of PGE-M, $PGE_2$ and creatinine.

In the *first* experiment, urine was collected the night before tumour transplantation and on the 6th and 8th days of tumour growth. Blood was also sampled on Days 6 and 8 for serum calcium, and on the 8th day only for PTH analysis; the animals were sacrificed. The test animals ($n = 7$) were treated orally at 08.00 and 20.00 with 0·8 mg of indomethacin/kg body weight. The concurrent controls ($n = 6$) were handled in the same way except that they received no drug.

In the *second* experiment, animals were divided randomly into 3 groups ($n = 8$): controls, indomethacin treatment (5 mg/kg/day, i.m.) and dexamethasone treatment (0·5 mg/kg/day, i.m.). Drugs were given at 08.00 and 20.00 beginning on the 5th day after tumour transplantation. These changes in the experimental design were necessary to prevent major side effects of indomethacin (*e.g.* intestinal ulceration and renal failure) and differences in tumour growth between the controls and dexamethasone-treated rats. At the end of this experiment indomethacin and dexamethasone serum levels were determined in the middle of the dosage interval. Tumour size was assessed by weight and by volume (V) which was calculated from the equation $V = \pi W^2 L/6$. Width (W) and length (L) were measured with calipers. In addition, tumour tissue was dissected immediately and fixed in buffered 4% formaldehyde solution, embedded in Paraplast[R] (Lancer, St Louis, Mo, U.S.A.) and 4–5μm sections were stained with haematoxylin–eosin.

*Prostaglandin analysis.*—The determination of urinary levels of PGE-M and $PGE_2$ was by quantitative mass spectrometry. $^3H$-labelled biosynthesized PGE-M (Seyberth *et al.*, 1976*b*) ($\sim 12 \times 10^4$ ct/min sp. act. 150 Ci/mmol) and 1 μg of 3,3,4,4-tetradeutero-$PGE_2$ together with $^3H$-labelled, synthetic $PGE_2$ ($\sim 15 \times 10^4$ ct/min; sp. act. 120–170 Ci/mmol) were added to 5 ml of rat urine and the pH was adjusted to 3·2 with formic acid. The ethyl acetate extract was concentrated and chromatographed on a 2g open silica-gel column (1cm i.d.) using the solvent system ethyl acetate: toluene (7:3). PGE-M and $PGE_2$ were eluted in 100 ml. After methylation with freshly prepared diazomethane, the material was subjected to high-performance liquid chromatography (HPLC) using a 10μm silica-gel column (Seyberth *et al.*, 1976*b*). Because no $^2H$-labelled PGE-M was present in the sample up to this preparative step, the radioactivity in the PGE-M peak of the HPLC chromatogram was determined, to assess the recovery of PGE-M. Subsequently eluates of PGE-M and $PGE_2$ were converted to the methoxime and trimethylsilylether derivatives. After adding 1 μg of $d_3$-methoxime derivative of the methylester of PGE-M to the PGE-M sample, the isotope ratios were determined with a gas chromatography–mass spectrometry (GC–MS) system.

*Miscellaneous procedures.*—Serum PTH concentration was determined by radioimmunoassay according to the procedure of Streibl *et al.* (1979). This method can detect serum PTH values in normal rats and can clearly discriminate between intact and parathyroidectomized rats (Minne *et al.*, unpublished). Serum concentrations of indomethacin were determined by the HPLC method of Skellern & Salole (1975). Serum concentrations of dexamethasone were also determined by HPLC: serum (500 μl) was diluted 1:1 with water, poured on to 2 g of Extrelut$^R$ (E. Merck, Darmstadt, W. Germany) and eluted with ethyl acetate (25 ml). The evaporated extract was reconstituted in eluent and chromatographed on a Porasil$^R$ column (Waters Associates, Milford, Mass., U.S.A.) with the eluent dichloromethane: methanol:glacial acetic acid (97:3:0·1); the detector was set at 254 nm. Prednisolone was used as the internal standard. Serum calcium concentrations were determined with a Corning 940 Analyser and the determinations of creatinine were performed with a Beckman Creatinine-Analyser II.

*Materials and equipment.*—All solvents were glass-distilled reagents from Prochem Company (Wesel, W. Germany). Unlabelled and tetradeuterated $PGE_2$ was generously provided by Dr U. Axen, The Upjohn Company (Kalamazoo, Mich., U.S.A.). Tritiated $PGE_2$ and 15-keto-13,14-dihydro $PGE_2$ (sp. act. 150 Ci/mmol) were obtained from Amersham Buchler (Braunschweig, W. Germany). Chemically synthesized PGE-M was donated by Drs J. R. Boot and N. J. A. Gutheridge, The Lilly Research Centre Ltd (Windlesham, England). $d_3$-Methoxyamine HCl was purchased from Serva (Heidelberg, W. Germany) and N,O-bis (trimethylsilyl) trifluoroacetamide from Fluka AG (Buchs, Switzerland) respectively.

Diazomethane was prepared as described previously (Sweetman *et al.*, 1973). Indomethacin and dexamethasone were gifts from Sharp and Dohme (Munich, W. Germany). The aqueous indomethacin sodium salt solution was prepared by neutralization of indomethacin with sodium carbonate. Prednisolone was provided by E. Merck (Darmstadt, W. Germany).

HPLC of prostaglandins was performed with two Waters Associates (Milford, Mass., U.S.A.) pumps (Model 6000 A) coupled to a solvent-flow programmer (Model 660). The silica-gel column was a prepacked 10μm Porasil column (Waters Associates). For mass-spectrometric analysis a Hewlett-Packard HP 5992 A microprocessor-controlled GC-MS system (Hewlett-Packard Company, Palo Alto, Calif., U.S.A.) equipped with a glass capillary column (Erlenmaier *et al.*, 1979) and working in the selected ion-monitoring mode was used.

*Statistical methods.*—Where appropriate, the results were subjected to an analysis of variance followed by analysis of co-variance and the Scheffé test. Otherwise the rank-sign sum test was applied.

### RESULTS

Five to 6 days after tumour transplantation, the Walker carcinosarcoma started to grow rapidly from an almost non-palpable tumour to a tumour of $7{\cdot}4 \pm 4{\cdot}4$ g (mean ± s.d.) in the untreated rats and $6{\cdot}4 \pm 1{\cdot}7$ g in the indomethacin-treated rats within 2 days. During that time hypercalcaemia was present in most rats and the urinary excretion of PGE-M rose about 3-fold (Fig. 1A). The animals with the highest PGE-M excretion rates were usually the ones with the highest serum calcium concentrations. However, the hypercalcaemia in the indomethacin-treated rats was in the same range as in the control group, despite a significant decrease of PGE-M excretion below the basal level (Fig. 1B). Serum concentrations of immunoreactive PTH were $3{\cdot}7 \pm 1{\cdot}2$ μl S.U.I./100 μl in the untreated rats and $3{\cdot}4 \pm 1{\cdot}0$ μl S.U.I./100 μl in the indomethacin-treated animals, which were not significantly different from those of untreated control rats ($3{\cdot}1 \pm 0{\cdot}6$ μl S.U.I./100 μl).

Pharmacological intervention in the second experiment was not started until the 5th day after s.c. injection of the ascites cells of the Walker carcinosarcoma. The drug concentrations in the middle of the dosage interval were $19{\cdot}2 \pm 2{\cdot}6$ $\mu g$ indomethacin/ml and $0{\cdot}143 \pm 0{\cdot}077$ $\mu g$ dexamethasone/ml. The effects of the drug treatments on the 8th day after tumour transplantation on urinary excretion of PGE-M and $PGE_2$, serum calcium concentration and tumour weight are shown in Fig. 2. The Walker carcinosarcoma-bearing rats excreted 2–3 times more PGE-M than normal rats. Indomethacin markedly suppressed the PGE-M excretion below the normal range, while dexamethasone reduced to only a small extent the increased urinary PGE-M excretion rate. Urinary $PGE_2$ excretion, a parameter of renal $PGE_2$ synthesis, was not significantly altered in the Walker tumour-bearing rats, indicating that mainly systemic PGE-production contributes to the elevated PGE-M excretion in these rats.

FIG. 1.—Time course of the rise in urinary PGE-M excretion and serum calcium concentrations in Walker carcinosarcoma-bearing rats: (A) no pharmacological intervention; (B) indomethacin treatment (0·8 mg/kg, orally twice a day) starting one day before tumour transplantation. The shaded area represents the normal range of serum calcium concentration. Values from each rat are identified by a separate symbol. All the compared means for PGE-M were significantly different at a level of $P < 0{\cdot}01$. CR = urinary creatinine.

FIG. 2.—Effects of indomethacin or dexamethasone on urinary PGE-M and $PGE_2$ excretion, on serum calcium concentration and on tumour weight in Walker tumour-bearing rats. Treatments (5 mg of indomethacin/kg/day or 0·5 mg of dexamethasone/kg/day i.m., respectively) were started 5 days after tumour transplantation and continued to the 8th day, when the animals were killed. Bars give the median for each group. Each rat is represented by a different symbol. The shaded area represents the mean $\pm$ 2 s.d. of 6 normal concurrent control rats. Cr = urinary creatinine.

Indomethacin almost completely abolished $PGE_2$ in urine, while dexamethasone did not significantly affect the $PGE_2$ excretion. Despite marked inhibition of systemic and renal PGE-production, indomethacin had no effect on serum calcium concentration. The normal serum calcium concentrations of 3 rats in the indomethacin group were associated with a relatively small tumour growth. In all 3 groups tumour size and volume were not significantly different, and there was no noticeable difference in tumour histology, such as invasion of inflammatory cells or necrobiosis.

Fig. 3 shows a plot of tumour weight against calcium concentration. Irrespective of the treatment, there is a close correlation between these two parameters. There was a similar relationship when tumour weight was replaced by tumour volume ($y = 0{\cdot}474x + 4{\cdot}54$; $r = 0{\cdot}725$). The lower serum calcium concentrations of dexamethasone-treated animals can also be seen in this figure: all the calcium values of the dexamethasone-treated rats lie below the general regression line.

FIG. 3.—Relationship between tumour weight and serum calcium concentration in the Walker tumour-bearing rats of Fig. 2: untreated ●; indomethacin-treated, ○; and dexamethasone-treated, △.

### DISCUSSION

Increased systemic PGE production was associated with growth of the Walker carcinosarcoma in both experiments. The tissue or organ which contributes most to this increased PGE turnover remains to be identified; the tumour, an activated immune system or the bone tissue are possibilities.

Two different doses of indomethacin failed to affect the hypercalcaemia in the tumour-bearing rats, despite marked inhibition of PGE production. By the criteria of a prostaglandin-mediated process (Needleman, 1978) one has to exclude E-prostaglandins and probably any other product of the cyclo-oxygenase pathway as the mediator of this hypercalcaemia. These findings do not corroborate those of Powles *et al.* (1973) who observed a decrease in the high serum calcium concentrations in rats bearing the Walker tumour when they treated these animals with a combination of two cyclo-oxygenase inhibitors, aspirin and indomethacin, beginning 3 days before tumour-cell injection. Although prostaglandin production had not been monitored in these studies, one may assume some inhibition of prostaglandin synthesis by this kind of treatment. However, there were some possibly relevant differences in the experimental design: first the method of injecting the tumour cells (intra-arterially *vs* s.c.) and second, possible differences in the genetic make-up of the tumour lines. Recently Spiro & Mundy (1979) reported in preliminary form that there is a close correlation between production of bone resorbing activity by different Walker carcinosarcoma cell clones and the release of immunoreactive E-prostaglandins into the medium. Therefore, bone metastases of some tumour lines may contribute to localized osteolysis in the Walker-bearing rats. However, as shown with our strain, factors unrelated to prostaglandins have to be considered as humoral mediators of the hypercalcaemia, such as a PTH-like compound or an osteoclast-activating factor (OAF).

Dexamathasone was chosen as a pharmacological intervention to differentiate between glucocorticoid-resistant,

PTH-mediated hypercalcaemia (Dent, 1962; Raisz *et al.*, 1972) and a highly glucocorticoid - sensitive, OAF - induced hypercalcaemia (Mundy, 1978; Mundy *et al.*, 1978) in addition to the intervention at a different level of prostaglandin synthesis. Although we have not measured OAF, it appears unlikely that this factor is the hypercalcaemic mediator, as intact prostaglandin synthesis is required for OAF production (Yoneda & Mundy, 1979). The small lowering effect of dexamethasone on serum calcium does not argue against a PTH-mediated hypercalcaemia, because the dexamethasone serum concentrations in the rats are similar to those which have a direct inhibitory effect on bone-cell function *in vitro* (Raisz *et al.*, 1972; Chen & Feldman, 1979). In addition, serum concentrations of immunoreactive PTH in the upper normal range in the hypercalcaemic rats support the PTH hypothesis, particularly when the antiserum in the radioimmunoassay may cross-react only in part with ectopically secreted PTH. Serum concentrations of immunoreactive PTH were inappropriately high for a hypercalcaemic state, and previous experiments have shown that indomethacin does not influence PTH-induced hypercalcaemia (Seyberth *et al.*, 1976*a*). However, for the final proof of a PTH-like compound as the mediator of the hypercalcaemia in the Walker tumour-bearing rat, studies with inhibitors of PTH production or with PTH antagonists will have to be performed. An alternative hypothesis to PTH-mediated hypercalcaemia is that the hypercalcaemia-inducing agent is a product synthesized *via* the lipoxygenase pathway of arachidonate metabolism; this pathway is not inhibited by indomethacin (Hamberg & Samuelsson, 1974). Only an earlier intervention in the metabolism of arachidonate, such as the inhibition of its release from phospholipids, would prevent the production of metabolites of the cyclo-oxygenase and lipoxygenase pathways. The small but concomitant decrease of calcium concentration and PGE-M excretion in the rats treated with dexamethasone suggests that a lipoxygenase product is the actual mediator of the hypercalcaemia, whilst the stimulated PGE turnover is an associated biochemical event, without any causal relationship to the pathogenesis of the hypercalcaemia. Unfortunately, as demonstrated in our experiments, dexamethasone does not appear to be an ideal pharmacological tool for manipulating prostaglandin production *in vivo*, though the serum concentrations of dexamethasone were higher than those which inhibit prostaglandin release *in vitro* (Tam *et al.*, 1977). Therefore, there is apparently still sufficient non-esterified arachidonic acid available, to be metabolized by lipoxygenase to a variety of hydroxy polyunsaturated fatty acids (Borgeat & Samuelsson, 1979; Murphy *et al.*, 1979). More effective and specific inhibitors of lipoxygenase, and more knowledge about the effects of these eicosonides on calcium and bone metabolism, are needed to exclude an eicosonide-mediated hypercalcaemia.

In conclusion, there is an increased PGE production in the Walker carcinosarcoma-bearing rat, which is unrelated to the paraneoplastic syndrome of hypercalcaemia. The humoral mediator remains to be identified. The only parameter closely correlated with the serum calcium concentration is tumour size.

The urinary excretion of PGE-M may be considered as a biochemical marker of tumour load, but not as an indicator of a prostaglandin-mediated hypercalcaemia in the Walker carcinosarcoma-bearing rat. This observation may be applicable also to certain human tumours, as recently shown in a breast cancer patient with increased production of immunoreactive $PGE_2$, in whom hypercalcaemia was unaffected by indomethacin treatment (Caro *et al.*, 1979).

We thank Mr H. Löhrke (Institut für Experimentelle Pathologie des Deutschen Krebsforschungszentrums, Heidelberg, W. Germany) for the Walker carcinosarcoma-bearing rats, Dr L. Nemeth for histological evaluation and Dr H. Scheurlen for his statistical calculations. This study was supported by grants from the Deutsche Forschungsgemeinschaft, Se 263–2/3, and Sonderforschungsbereich 87 Endokrinologie.

### REFERENCES

Borgeat, P. & Samuelsson, B. (1979) Arachidonic acid metabolism in polymorphonuclear leukocytes: Unstable intermediate in formation of dihydroxy acids. *Proc. Natl Acad. Sci. U.S.A.*, **76**, 3213.

Caro, J. F., Besarab, A. & Flynn, J. T. (1979) Prostaglandin E and hypercalcemia in breast carcinoma: Only a tumor marker? *Am. J. Med.*, **66**, 337.

Chen, T. L. & Feldman, D. (1979) Glucocorticoid receptors and actions in subpopulations of cultured rat bone cells. *J. Clin. Invest.*, **63**, 750.

Dent, C. E. (1962) Some problems of hyperparathyroidism. *Br. Med. J.*, ii, 1419.

Erlenmaier, T., Müller, H. & Seyberth, H. W. (1979) Combined capillary column gas chromatography–mass spectrometric method for the quantitative analysis of urinary prostaglandins. *J. Chromatogr.*, **163**, 289.

Flower, R.J. (1974) Drugs which inhibit prostaglandin biosynthesis. *Pharmacol. Rev.*, **26**, 33.

Frölich, J. C., Wilson, T. W., Sweetman, B. J. & 5 others (1975) Urinary prostaglandins. Identification and origin. *J. Clin. Invest.*, **55**, 763.

Green, K. (1971) Metabolism of prostaglandin $E_2$ in the rat. *Biochemistry*, **10**, 1072.

Hamberg, M. (1972) Inhibition of prostaglandin synthesis in man. *Biochem. Biophys. Res. Commun.*, **49**, 720.

Hamberg, M. & Samuelsson, B. (1974) Prostaglandin endoperoxides. Novel transformations of arachidonic acid in human platelets. *Proc. Natl Acad. Sci. U.S.A.*, **71**, 3400.

Lands, W. E. M. (1979) The biosynthesis and metabolism of prostaglandins. *Ann. Rev. Physiol.*, **41**, 633.

Minne, H., Raue, F., Bellwinkel, S. & Ziegler, R. (1975) The hypercalcaemic syndrome in rats bearing the Walker carcinosarcoma 256. *Acta Endocrinol. (Kbh.)*, **78**, 613.

Minne, H., Ziegler, R. & Arnaud, C. D. (1978) Paraneoplastic parathyroid hormone production by the hypercalcemic Walker carcinosarcoma 256 of the rat. In *Endocrinology of Calcium metabolism.* Eds Copp & Talmage. Oxford: Excerpta Medica. p. 385.

Mundy, G. R. (1978) Calcium and cancer. *Life Sci.*, **23**, 1735.

Mundy, G. R., Rick, M. E., Turcotte, R. & Kowalski, M. A. (1978) Pathogenesis of hypercalcemia in lymphosarcoma cell leukemia. *Am. J. Med.*, **65**, 600.

Murphy, R. C., Hammarström, S. & Samuelsson, B. (1979) Leukotriene C: A slow-reacting substance from murine mastocytoma cells. *Proc. Natl Acad. Sci. U.S.A.*, **76**, 4275.

Needleman, P. (1978) Experimental criteria for evaluating prostaglandin biosynthesis and intrinsic function. *Biochem. Pharmacol.*, **27**, 1515.

Powles, T. J., Clark, S. A., Easty, D. M., Easty, G. C. & Neville, A. M. (1973) The inhibition by aspirin and indomethacin of osteolytic tumour deposits and hypercalcaemia in rats with Walker tumour, and its possible application to human breast cancer. *Br. J. Cancer*, **28**, 316.

Raisz, L. G., Trummel, C. L., Wener, J. A. & Simmons, H. (1972) Effect of glucocorticoids on bone resorption in tissue culture. *Endocrinology*, **90**, 961.

Seyberth, H. W. (1978) Prostaglandin-mediated hypercalcemia: A paraneoplastic syndrome. *Klin. Wochenschr.*, **56**, 373.

Seyberth, H. W., Hubbard, W. C., Oelz, O., Sweetman, B. J., Watson, J. T. & Oates, J. A. (1977) Prostaglandin-mediated hypercalcemia in the $VX_2$ carcinoma-bearing rabbit. *Prostaglandins*, **14**, 319.

Seyberth, H. W., Raisz, L. G. & Oates, J. A. (1978) Prostaglandins and hypercalcemic states. *Annu. Rev. Med.*, **29**, 23.

Seyberth, H. W., Segre, G. V., Hamet, P., Sweetman, B. J., Potts, J. R., Jr & Oates, J. A. (1976*a*) Characterization of the group of patients with the hypercalcemia of cancer who respond to treatment with prostaglandin synthesis inhibitors. *Trans. Assoc. Am. Physicians*, **89**, 92.

Seyberth, H. W., Sweetman, B. J., Frolich, J. C. & Oates, J. A. (1976*b*) Quantification of the major urinary metabolite of the E prostaglandins by mass spectrometry: Evaluation of the method's application to clinical studies. *Prostaglandins*, **11**, 381.

Skellern, G. & Salole, E. G. (1975) High-speed liquid chromatographic analysis of indomethacin in plasma. *J. Chromatogr.*, **114**, 483.

Spiro, T. P. & Mundy, G. R. (1979) Prostaglandins mediate *in vitro* bone resorption caused by cultured Walker rat 256 carcinosarcoma cells. *1st Ann. Meeting Am. Soc. Bone Mineral Res.* (Abstr.).

Streibl, W., Minne, H., Raue, F. & Ziegler, R. (1979) Radioimmunoassay for human parathyroid hormone for differentiation between patients with hypoparathyroidism, hyperparathyroidism and normals. *Horm. Metab. Res.*, **11**, 375.

Sweetman, B. J., Frölich, J. C. & Watson, J. T. (1973) Quantitative determination of prostaglandins A, B and E in sub-nanogram range. *Prostaglandins*, **3**, 75.

Tam, S., Hong, S. L. & Levine, L. (1977) Relationships, among the steroids, of anti-inflammatory properties and inhibition of prostaglandin production and arachidonic acid release by transformed mouse fibroblasts. *J. Pharmacol. Exp. Ther.*, **203**, 162.

Tashjian, A. H., Jr (1978) Role of prostaglandins in the production of hypercalcemia by tumors. *Cancer Res.*, **38**, 4138.

Tashjian, A. H., Jr, Voelkel, E. F. & Levine, L. (1977*a*) Effects of hydrocortisone on the hypercalcemia and plasma levels of 13,14-dihydro-15-keto-prostaglandin $E_2$ in mice bearing the $HSDM_1$ fibrosarcoma. *Biochem. Biophys. Res. Comm.*, **74**, 199.

Tashjian, A. H., Jr, Voelkel, E. F. & Levine, L. (1977b) Plasma concentrations of 13,14-dihydro-15-keto-prostaglandin $E_2$ in rabbits bearing the $VX_2$ carcinoma: Effects of hydrocortisone and indomethacin. *Prostaglandins*, **14**, 309.

Yoneda, T. & Mundy, G. R. (1979) Monocytes regulate osteoclast-activating factor production by releasing prostaglandins. *J. Exp. Med.*, **150**, 338.